# Content of clinicians’ communication with patients suffering from spinal pain in assessment situations in a specialized spine center: A qualitative study evaluating psychologically informed pain assessments before and after clinicians’ participation in an Acceptance and Commitment Therapy course

**DOI:** 10.1186/s12891-023-06392-z

**Published:** 2023-07-04

**Authors:** Sophie Lykkegaard Ravn, Tonny Elmose Andersen, Berit Schiøttz-Christensen

**Affiliations:** 1 Specialized Hospital for Polio and Accident Victims, Fjeldhammervej 8, 2610 Roedovre, Denmark; 2 Department of Psychology, University of Southern Denmark, Campusvej 55, 5230 Odense M, Denmark; 3 Research Unit of General Practice, University of Southern Denmark, J. B. Winsløws Vej 9A, 5000 Odense C, Denmark

**Keywords:** ACT, Cognitive behavioral, Spinal pain, Assessment, Chronic pain, Communication

## Abstract

**Introduction:**

Assessment is an important part of chronic pain rehabilitation and should be conducted in line with the current biopsychosocial conceptualization of pain to capture the subjectivity and context of pain. However, pain assessment is commonly conducted from a biomedical framework. A course in Acceptance and Commitment Therapy (ACT) was provided to spinal pain clinicians as a framework to promote more person-centered and psychosocially focused assessments and related psychologically informed practices. The purpose of this qualitative study was to explore the verbal content of clinicians’ communication with patients experiencing spinal pain in assessment situations before and after clinicians participated in an ACT course.

**Methods:**

Pain assessments of patients with chronic low back pain conducted by six spinal pain clinicians from different professions were audio-recorded and transcribed. This was done before and after participation in an eight-day ACT course with four following supervisions. A thematic analysis was carried out by two authors across all material, and a comparison of the applied number of codes pre-course and post-course was carried out as an indicator of change.

**Results:**

Data consisted of transcripts from the six clinicians across 23 different patients (12 before course participation). Through analysis, 11 codes were developed, which were clustered in three overarching themes: Psychological domains, Communication Techniques, and Intervention Elements. Overall, there was an increase in the application of many of the codes in the transcripts from pre-course to post-course, however with large differences across codes. Increases were primary related to the discussion of life values and value-based action and quality of life as well as the employment of mirroring, challenging beliefs and assumptions, and addressing coping and pacing.

**Conclusions:**

While not the case for all factors, the present findings indicate an increase in including psychological factors and employing interpersonal communication skills after a course in ACT. However, it remains unknown due to the design if the changes reported in this study reflect a clinically valuable change and whether they are due to the ACT training itself. Future research will improve our understanding of the effectiveness of this type of intervention in assessment practices.

## Introduction

Assessment is an integrated part of pain treatment and rehabilitation and is the foundation for ensuring optimal intervention and management [1]. The current biopsychosocial conceptualization of pain among others includes a strong focus on subjectivity and patient experiences [2]. This implicates a more person-centered clinical approach with a focus on psychological and social elements, which should also be reflected in clinical assessment encounters in addition to biomedicalelements. However, this is often not the case.

While psychologically informed pain practice is getting increased attention [3], many patients with chronic pain struggle to be regarded and understood as a person in the health care system [4], and assessment encounters often rely heavily on biomedical and quantitative features, as for example mentioned by Dansie and Turk [5]. A part of the explanation for the lack of inclusion of psychosocial elements may be the perceived barriers of the clinicians [6, 7]. Wideman and colleagues [8] recently raised the question of how the inherent subjectivity of pain can be optimally integrated in assessment and argued for the importance of a multimodal assessment model. Such an approach entails a shift in content to, among others, the patient’s qualitative pain narrative, but also a shift in methods to elements such as talking and listening [8], thereby also underlining the importance of interpersonal communication skills. In this line of work, it is therefore important to take all aspects of pain and the person living with pain into consideration. This is in line with the perspective of subjective medicine with its focus on patients’ lives rather than solely on patients’ bodies [9] and correspond well with the overall perspective of many psychological therapies, among others Acceptance and Commitment Therapy (ACT).

ACT is a third-wave cognitive behavioral therapy that focuses on the promotion of functioning and life quality rather than symptom control and reduction and has an embedded emphasis on values, context, and subjectivity [10, 11]. ACT has in recent years been explored as framework for psychologically informed health interventions among a broad range of health professionals as well as lay people (for a systematic review, see [12]). Like many other therapeutic traditions, it also incorporates some core therapeutic and communicative principles such as for example active listening and validation of emotions. In the present study, ACT was chosen for these reasons and due to its focus on value-based actions, encouraging people to do what matters to them despite pain.

Authentic exploration of how pain affects the life of the patient and what matters most in the patients’ life are important communicative practices requiring training. However, it remains unknown if ACT training could provide such a shift in assessment practices in non-mental health pain professionals. In the present project, ACT was therefore chosen as a framework for such training to promote assessments focusing on broader and psychological themes as well as applying related psychologically informed practices.

The purpose of this study was to explore the change of verbal communication by clinicians with patients suffering from spinal pain in assessment situations before and after ACT training.

## Methods

### Study setting

The present study took place in a specialized regional spine center in Region of Southern Denmark, which supports around 1.2 million inhabitants. The primary purpose of the spine center is to assess patients with spinal pain and to refer to relevant treatment if needed. To be referred to the spine center, patients must suffer from ongoing and disabling pain (> 12 weeks) that they have received previous treatment for in primary sector. As a part of a project focused on improving patients’ quality of life and ability to take care of their own life situation, a team of spinal pain clinicians working at the spine center participated in an ACT course. The course was specifically designed for the short assessment contact with spinal pain patients as is the everyday reality at the spine center. The course was carried out with two ACT therapists and supervised by a certified ACT psychologist. The course consisted of two 2-day seminars and four full day courses over the course of five months continued by four 2-h sessions of supervision over the following months. For the purpose of the present qualitative study, data was collected from clinicians before and after participating in the course. Time between data collection pre-course and data collection post-course were approximately seven months, and time between end of training and post-course data collection was approximately three months, in which time the clinicians received supervision.

### Study participants and data collection

A total of six spinal pain clinicians with expertise in managing patients with low back pain participated in the present study. Further information can be seen in the Result Section of this paper. Data consisted of audio recordings of the clinicians’ initial assessment consultations with different patients with spinal pain at the spine center. These assessments took around 30 min and were intended to evaluate the patient and to inform potential treatment. In most cases, the patient and the clinician were alone, but in one case a translator was present, in four cases patients were accompanied by a relative, and in four cases additional clinicians were present a part of the session. In one of these cases, an additional clinician was present for the first short part of the assessment (only speaking a few sentences), and in two other cases, an additional clinician was present in the latter part of the sessions. In the fourth case, two additional clinicians entered the sessions in the latter part. In all four cases, the dialogue was dominated by the clinician that was a part of the present study. Every clinician had to provide two recordings from both pre-course and post-course (i.e., each clinician had to provide data from four different patients).

### Data analysis

The assessment encounters as well as the discussions in the analytical process were in Danish. The audio recordings were transcribed in a verbatim manner prior analysis by a research secretary in Danish, who knew both the population and the clinical terms. For publication purposes, citations were translated from Danish to English by SLR. In this process, sentence structure was mildly adapted to be meaningful in English.

The first step of the analyses was overall familiarization with data. The analysis was a two-step procedure. First, a thematic analysis was carried out. Then, calculations of the number of applied codes were carried out. These two steps are outlined below.

Thematic analysis was applied as inspired by Braun and Clarke [13, 14] to identify psychologically informed content and approaches in the dialogues. The approach was inspired by a combination of what Braun and Clarke [14] refers to as a reflexive approach and a codebook approach. This means that a reflective, organic approach of coding was applied to identify patterns in the material, but with two raters who discussed codes (i.e., coding consensus) and a shared coding tree [14]. The thematic analysis was carried out by SLR and TEA, who are psychologists of educational background and have experience from the area of chronic pain and different cognitive and behavioral therapies, including ACT. Both also have prior experience with conducting qualitative research. Data was coded independently by SLR and TEA one transcript at a time. After coding a transcript, they met to compare and discuss the coding for the given transcript. In doing this, they also developed a shared coding tree to be applied and further developed along the way. There was a shared understanding of ACT as framework prior to commencing the analysis, but data was coded inductively. Hence, the data analysis was grounded in the data material, but was not analyzed using a specific theoretical lens nor a pre-existing coding framework [14]. Codes were used as an analytical tool to highlight more narrow elements in the data relevant for our research question [14]. Both explicit examples of codes as well as more latent expressions of codes were coded [13, 14]. In this coding process, there was a focus on dialogue initiated by the clinicians. After coding all transcripts, the list of codes was used as a basis to develop overall, multifaceted themes across the codes. In this study, themes were understood as themes of shared topics rather than shared meaning [14]. This was done with check-backs to data to ensure that all relevant data was coded. Here, some adjustments were made to the coding, and the material was looked through again. In this process, the developed themes were critically discussed several times. SLR returned to the material afterwards to ensure proper usage of the coding framework and discussed specific issues with TEA. Minor changes were carried out at this point. Hence, this work was a continuous process, in which the authors met again and again to discuss the material and refine the coding. The material (whether it was pre-course or post-course) was coded in the same manner. This process gave a full overview of the material. This part of the analysis is what is referred to as the thematic analysis, which gave insights into the thematic content and patterns of the assessments. A narrative synthesis of the codes and themes are presented in this paper with examples from the material. This serves as the first part of the analysis.

After the thematic analysis, the second part of the analysis was carried out. Here, SLR and TEA compared the frequency of the different codes (i.e., the number of times each code was applied) in the pre-course and post-course material and calculated percentual change. This was used as a frequency indicator of different psychologically informed elements based on the assumption that each code indicated a paragraph of a psychologically informed elements. Hence, an increase in codes could be used as an indicator of increase of psychologically informed content and practices. Therefore, this was done as an additional analytical step to gain insight into potential changes in content before and after the course.

### Ethical approvals

The main study was approved by the Danish Data Protection Agency (file no. 1–16-02–477-16). According to the Health Research Ethics Committee of Region of Southern Denmark, the study did not need any further approval (file. no. 150/2016). Clinicians and patients gave written informed consent to have the assessments audio recorded and that this material could be used for research purposes.

## Results

### Descriptive characteristics

A total of six clinicians from different professions participated in the study: One chiropractor, one medical doctor, two physiotherapists, and two nurses. The clinicians’ mean age was 48 years, and 83.3% were women. They had a mean of 12.5 years’ experience working with low back pain and had a minimum of 6 years’ experience at the Spine Center before the ACT course (mean 7.3 years). Five clinicians provided two assessments both pre-course and post-course (*n* = 20), and one clinician provided two pre-course and one post-course (*n* = 3), leaving a total of 23 transcribed clinical assessments encounters. The patients in these clinical assessment encounters were patients (both women and men) with ongoing low back pain referred to assessment at the spine center and were between 18 and 60 years of age.

### Thematic analysis and counts of applied codes

**Fig. 1:**
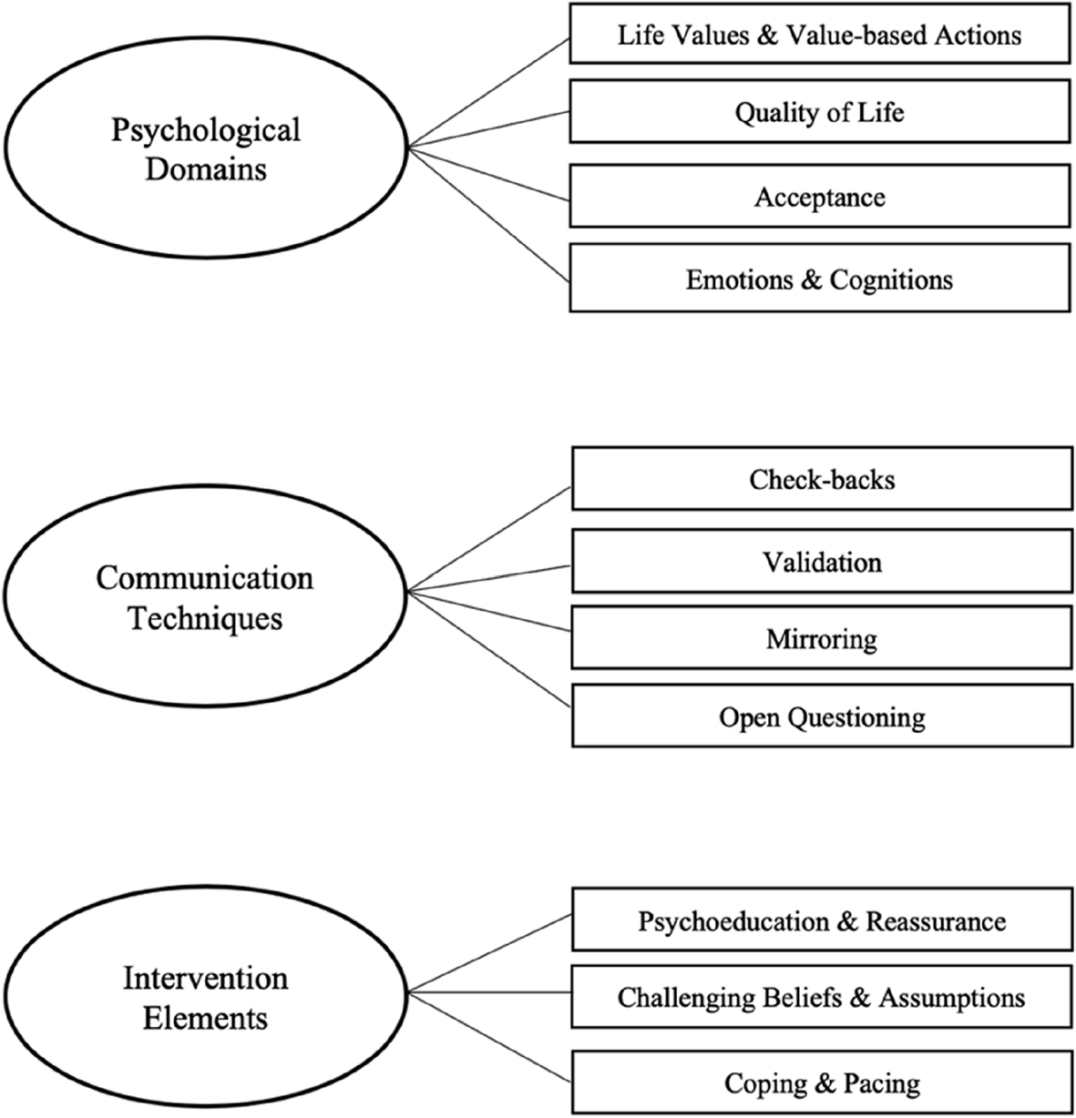
Visual overview of the three overarching themes (visualized in circles) and the related codes (visualized in squares)

Through the analysis, we coded the data using a total of 11 codes, which were clustered in three overarching themes: Psychological domains, Communication Techniques, and Intervention Elements (Fig. 1). These codes and themes underlined the content of the assessments.

**Fig. 2:**
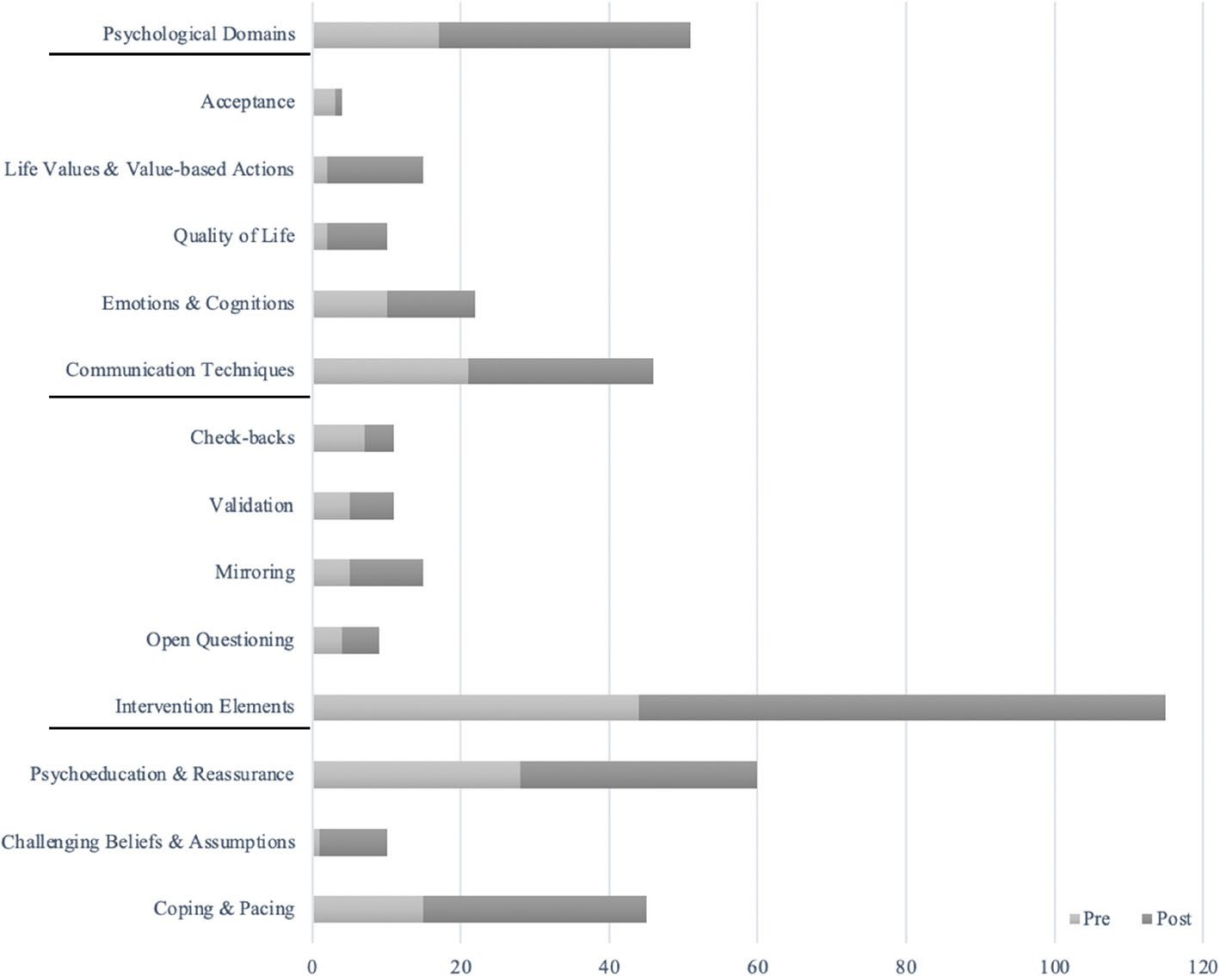
Visual overview of number of codes applied in pre-course and post-course material (both for the overall themes and for the 11 codes clustered under each theme)

Further, we also explored differences in content in the pre-course material compared to the post-course material by comparing the number of applied codes in the analysis. Overall, the three themes were more present in the post-course material compared to the pre-course material, however in varying degree (Table 1, Fig. 2). Further, there were large variations in the application of the different codes under each theme when comparing them in the pre-course material and the post-course material. Some codes were applied more, others were used similarly, and others again were applied less. There were also variations in the number of applied codes from transcript to transcript (both across and within clinicians). In the pre-course material, a mean of 6.5 (range 1–20) codes were applied pr. transcript. In the post-course material, a mean of 11.7 (range 4–24) codes were applied pr. transcript.

**Table 1:** Number of applied codes under each theme in pre-course and post-course material as well as percentual increase

Theme	Pre-Course Material	Post-Course Material	Percentage Change
**Psychological domains**	**17**	**34**	** + 100%**
Life Values & Value-based Action	2	13	+ 550%
Quality of Life	2	8	+ 300%
Acceptance	3	1	- 66.66%
Emotions & Cognitions	10	12	+ 20%
**Communication Techniques**	**21**	**25**	** + 19.05%**
Check-backs	7	4	- 42.86%
Validation	5	6	+ 20%
Mirroring	5	10	+ 100%
Open Questioning	4	5	+ 25%
**Intervention Elements**	**44**	**71**	** + 61.36%**
Psychoeducation & Reassurance	28	32	+ 14.29%
Challenging Beliefs & Assumptions	1	9	+ 800%
Coping & Pacing	15	30	+ 100%

The content of the different codes and the variations in code application in the pre-course and post-course material are unfolded per theme below.

#### Psychological domains

Exploration of several psychological domains were identified in the transcripts. These were coded as: *Life Values & Value-based Actions, Quality of Life, Acceptance*, and *Emotions & Cognitions.* The code of *Life Values & Value-based Actions* was used for dialogue about important life elements and how this informed behavior and actions in everyday life. This was broadly defined to also include broader questions or discussions of everyday life functioning, but did not include more instrumental, symptom specific functioning questions. This was, for example, illustrated in the following questions:

*“So what is actually important to you, because you are doing many things, so if you had to think about what is important to you?”* (Clinician 1)

*“So what obstructs you in doing all the things you would like to?”* (Clinician 1)

*“What would it mean to you to find a solution to this? What difference would it make in your life?”* (Clinician 3)

*”How does this bother you in your everyday life?”* (Clinician 4)

*“ (..) Can you adjust in this so you still do some of the things that are important to you (..)?”* (Clinician 5)

*“What is it that is important for you to be able to do?”* (Clinician 6)

Further, the code *Quality of Life* was applied for explicit questions about the patients’ perceptions of their quality of life as well as more implicit open questions about for example their general well-being or how a good everyday life looks like. This was, for example, asked in the following ways:

*“What does in fact give you quality of life?”* (Clinician 1)

*“So a good everyday life to you, what is that?”* (Clinician 1)

*“So are you satisfied with it as it is now? Well, you can say (..) that you have these challenges, but are you satisfied with your life as it is?”* (Clinician 1)

*“How does this affect your quality of life?* (Clinician 2)

The third code, *Acceptance,* was used for explicit communication about acceptance of life circumstances or learning to live with it. The final code in this theme, *Emotions & Cognitions,* was broadly defined and was used to any communication about emotional and cognitive elements. This could for example be direct questions about how the pain or the situation made the patients’ feel emotionally but could also be more implicit discussions or descriptions of the patients’ thoughts, expectations, and feelings. This did not include simple “what do think about this idea”-questions. Rather, this code is, for example, illustrated in the following quotes by Clinician 3:

*“How do you feel about this?*”

*“How do you respond mentally, emotionally (..)?”*

In addition to the above-outlined content of the first theme, we also explored the differences in the application of codes in pre-course material compared to post-course material. Overall, there was a clear increase from pre-course to post-course in this theme. In the pre-course assessments, the codes in this theme were applied 17 times, while they were applied 34 times in the post-course material, hence a 100% increase (Table 1). However, the level of application varied markedly between specific codes. The codes of *Life Values & Value-based Action* and *Quality of Life* were applied considerably more post-course compared to pre-course, while *Emotions & Cognitions* only was applied a little more, and *Acceptance* was applied less in the post-course compared to pre-course material. For visual overview, please see Fig. 2.

#### Communication techniques

Several communication techniques were applied in the assessments. These were coded as: *Check-backs, Validation, Mirroring,* and *Open Questioning. Check-backs* was used to code clinicians’ efforts to verify the information given by the patients and thereby attempt to ensure a correct understanding, but also as a means to include the patient by asking for example “What do you think about this?” and “Is this something that makes sense?”. *Validation* was used to code expressions from the clinicians that aimed to recognize and show understanding of the patients’ thoughts, feelings, or behaviors. This was often used in cases were the patient described or signaled being fearful or insecure, either explicitly or implicitly. This was generally by explicitly stating that their worries or conceptions were understandable and normal. The code of *Mirroring* was applied to clinicians’ efforts to reproduce key parts of what the patients have said or done in a manner aimed to ensure the patients’ insights, but also explicitly statements in which the clinician was letting them know that they understood and seen. *Mirroring* was, for example, used in cases where the clinicians explicitly used the patient’s reactions or responses. This was for example in cases, where the clinician said:

*“There you see, now you glow much more.”* (Clinician 1)

*“I can see this makes you happy.”* (Clinician 1)

*“I can hear this is also important.”* (Clinician 3)

The final code in this theme*, Open Questioning,* was used to code open and broad questions about more person-centered, everyday life elements and not symptom nor activity specific elements. Generally, the clinicians were good at asking (open) questions, but these were often related to specific symptoms or activities. While this is also an important part of the assessment encounter, it was not considered a part of the developmental work in the present project and was therefore not coded. Follow-up questions as a natural part of a dialogue such as “what do you do then” were also not coded. This code was also used in cases where people had an emotional response in the room to open for a more emotionally focused dialogue – for example by asking the patient what they thought of their emotional reaction.

For the overall theme, there was a small increase in the application of these techniques from pre-course to post-course. Specifically, the codes in this theme were applied 21 times in the pre-course material, while they were applied 25 times in the post-course material, hence a 19.05% increase (Table 1). For the specific codes, it was only the amount of mirroring that clearly increased from pre-course to post-course. The number of check-backs were less at post-course compared to pre-course, while the use of validation and open questioning were almost the same with a small increase. For visual overview, please see Fig. 2.

#### Intervention elements

Discussion of several intervention elements related psychologically informed assessment practice were identified in the transcripts. These were coded using four codes: *Psychoeducation & Reassurance, Coping & Pacing,* and *Challenging Beliefs & Assumptions.* The code *Psychoeducation & Reassurance* was used for the transfer knowledge to enable patients to improved coping or for the presentation of professional knowledge or experience to lower or remove the patients’ fears or doubts. While psychoeducation and reassurance are two different things, we found that the application of psychoeducation in the material implicitly aimed to reassure the patients. Of note, patient education of more biomedical oriented factors, which the clinicians were generally skilled in, was not coded unless it was evident that it was a part of reassuring the patient. The code is, for example, illustrated in the following quote from Clinician 6:

Clinician: [Have educated about the back and pain]. *“Can I borrow you hand? Look, now I can move your wrist, because you are relaxed, right? Try now to make a fist. Now, I cannot move the hand. What is most pleasant?”*

Patient: “*It is when I relax*.”

Clinician: “*It is. If one did this for – what do I know – a few years*?”

Patient: “*Then one would be stiff.”*

Clinician: “*It would be a little exhausting and stiff, right?”*

The next code, *Coping & Pacing* was used in which coping and/or pacing strategies were discussed. This included dialogue about activity balance, functionality, and management strategies. This was for example illustrated in the following quotes:

*“So, how do you manage right now when you have these challenges with…?”* (Clinician 1)

*“Could you imagine turning a little down for some of all the activities you have?”* (Clinician 1)

*”Do you have the possibility to lower the strain at your work?”* (Clinician 2)

*“What can you then do to ease it, when you feel like that?”* (Clinician 2)

*“How do you handle your pain in your everyday life?”* (Clinician 3)

*”But can we make it more optimal in your everyday life so your pain will be handled in a better way, so you in fact will experience less pain?”* (Clinician 5)

*”Can you do things differently?”* (Clinician 5)

Further, the final code, *Challenging Beliefs & Assumptions,* was used in cases where clinicians attempted to challenge the beliefs, assumptions, and perceptions of the patients – for example about the possibility to get pain free for example by talking to the patient about how realistic it was. In challenging beliefs and assumptions, the clinicians also often asked a series of questions like “*What happens if you do not do this*?” and “*Why is this important?*”. A specific example of such a dialogue can be seen in the following text from Clinician 6:

Clinician: *“Do you think it is realistic to go to work without pain? Pain free?”*

Patient: *“Yes. (..) I hope so. I have to say so because this was my hope.”*

Clinician:* “This makes me ask whether you need to be completely pain free before it is possible to be able to manage your work? (..) Or if this is the dream scenario?”*

Patient: *“It has to be comfortable to get up and go to work, so one can endure it.”*

Clinician: *“So one can endure it. To me, this is something a little else than being pain free, isn’t it?”*

Further, we also counted and compared the number of applied codes in the pre-course material and the post-course material. For the overall theme, there was a large increase in the application of these elements from pre-course to post-course. In the pre-course assessments, the codes in this theme were applied 44 times, while they were applied 71 times in the post-course material, hence a 61.36% increase (Table 1). All codes were used more post-course compared to pre-course, but with the largest differences in *Challenging Beliefs & Assumptions* and *Coping & Pacing*. For visual overview, please see Fig. 2.

#### Additional learning points

In addition to the above analysis, we also found some examples in the data material that may hold additional learning points, even though it is not coded due to the lack of correspondence with the study aim. In at least one situation, a probable attempt to reassure the patient ended up provoking the patient instead. Also, while the clinicians were generally good in asking follow-up questions, we saw some examples where the patient mentioned something that was (emotionally) challenging for them, which was not followed up by the clinician.

## Discussion

### Summary of findings

The present qualitative study aimed to explore the content of clinicians’ communication with patients with spinal pain in assessment situations in a specialist spinal pain setting before and after clinicians participated in an ACT course to explore its impact on communication content. The ACT course was used as a framework to change the conversation from a biomedical focus to a biopsychosocial focus.

First, we focused on the application of psychosocial domains and related psychologically informed practices across all material. Through the analytical process, we found a total of 11 codes clustered in three themes: i) Psychological domains, ii) Communication techniques, and iii) Intervention elements. With this, we found that even though the content of the assessments was still widely focused on biomedical features, there was also some important psychological domains and psychologically informed techniques that were included and employed. This illustrates some of the content that meaningfully can be included into assessment practices in such a setting.

Next, we focused on the differences in the application of codes in the pre-course and the post-course material. For all three themes, there was an increase in the application from pre-course to post-course. Looking at the specific codes, there were positive changes in 9 of 11 codes from pre-course to post-course. However, the magnitude of these increases was very different. While there was only a small difference over time in the second theme, Communication Techniques, there were large total increases in the two remaining themes of Psychological domains and Intervention Elements. Further, there were large variations in the applications of the specific codes within the themes. For about half the codes, a noticeable difference in application was identified from pre-course to post-course. Here, the five codes of *Life Values & Value-based Action, Quality of Life, Mirroring, Psychoeducation & Reassurance, Challenging Beliefs & Assumptions,* and *Coping & Pacing* were addressed considerably more post-course compared to pre-course. The four codes of *Emotions & Cognitions, Validation, Psychoeducation & Reassurance,* and *Open Questioning* was used a little more in post-course material compared to pre-course, but around the same level, while the remaining codes of *Acceptance* and *Check-backs* were used less in the post-course material compared to the pre-course material. Hence, the overall findings indicate a positive change, with some differences across codes, from pre to post course using the pragmatic design embedded in the clinic. While it is not possible to draw any definitive conclusions on the potential effect of the course due to the design, this may indicate a larger awareness and curiosity in the assessments on some of these elements.

### Discussion of empirical studies

ACT has become of increasing interest in chronic pain rehabilitation [15, 16], not only as a psychotherapeutic treatment modality, but also as a part of other parts of the rehabilitation process for example in physiotherapy [17]. Indeed, a recent systematic review concluded that ACT interventions can successfully be delivered by both non-mental health professionals and lay people [12]. While the present study is not testing an ACT intervention, the findings are in line with this, as is shows that non-mental health professionals can adopt and apply psychologically informed practices based on an ACT training course. Other studies in the area have used other psychologically informed approaches to gain similar effects on communication. A recent study in physiotherapists working with low back pain patients for example found that invalidating responses decreased and validating responses increased after participation in a course in brief cognitive functional therapy [18]. While we in the present study did not focus on invalidating communication, we also found a small positive change in validation from pre to post course. Also, a recent Swedish study used ACT as a framework for group education of interdisciplinary health care staff and reported reductions in patient sick leave [19], indicating that ACT training of interdisciplinary health care staff may improve patient outcomes. While the potential effects on patient outcomes remain unknown for the present study, it underlines the clinical relevancy of ACT in the context on interdisciplinary pain teams.

### Discussion of clinical perspectives

As an important part of the present study, there are some elements related to clinical and future perspectives that are important to discuss. As outlined above, the current conceptualization underlines pain as a biopsychosocial and multidimensional phenomenon [2]. In the present study, ACT was chosen as a framework to embed this broader understanding of pain and people in pain in clinical assessment encounters, as opposed to a more traditional, symptom focused biomedical approach [20, 21]. A successful assessment in line with the biopsychosocial model relies on therapeutic and communicative skills and the exploration of patients’ beliefs and concerns. In this way, the clinical assessment encounter has a lot in common with the therapeutic relationship in psychotherapy, which is acknowledged as fundamental for change across psychotherapeutic traditions [22]. We argue that this requires a shift in assessments from understanding pain only as a condition of the body to a condition of the person. This is needed to ensure a complete understanding of the patient’s condition and situation [23]. This is in line with the perspectives of many psychotherapies, among others ACT.

While the broader implementation of ACT is not necessarily without challenges [17], it holds some interesting perspectives for future work in this area, which is in line with for example recent work from Wideman et al. [8]. They argued for the importance of multidimensionality and multimodality in the assessment of pain as well as a more comprehensive and compassionate approach [8]. However, rather than focusing on specific therapeutic traditions such as ACT to achieve this, there are many ways to move forward to a more psychologically informed and biopsychosocial assessment practice. While we do find that ACT holds merit as a broader framework in this line of work, we argue that it is most important to move away from a symptom only focus to a broader focus on the whole patient and the surrounding context, of which psychological elements are highly important. This includes a curious, subjective approach to the patient. While ACT have some strengths for example with its focus on life quality and value-based behavior [10], other training courses could also have been provided as the chosen framework. This could for example be training of interpersonal communication skills or training in motivational interviewing [24]. Motivational interviewing has gained increasing attention in the area of chronic pain [25], including training in asking open questions, using reflective and empathic listening, being explorative, and supporting self-efficacy. This may be relevant to consider in future development of such assessment practices.

Another reflection from the present work is that future work in such practices is likely to benefit from a very specific aim and specific supervision afterwards. An important part of this is to consider why such a transformation is wanted, but also to reach agreement on how it specially look like in practice Spinal pain clinicians in assessment encounters are not psychotherapists, but rather need to be explorative on all life domains. Here, contextual factors – such as a short time frame – are also important to consider. This was aconsideration in the present study but is important to highlight and potentially further develop. It may also be beneficial to agree to some target areas that all clinicians should get around in their assessments – for example that of values – but also to agree on what to do with this information and how to use it to an individual, targeted approach.

A last and very important point is that of clinical value. The present work did not enable us to establish whether the differences we found had any clinical relevance for neither clinicianexperiences nor patient experiences nor outcomes. Clinical value ought to be an important part of future work in this area, also including the patient perspective in order ensure assessments practices are valuable, relevant, and meaningful to the patients.

### Limitations

While there are several strengths in the present study such as embedment in a clinical setting, there are also some important discussion points and limitations to take into consideration in interpretation of the results.

First, some limitations exist related to the design of the study. These are natural consequences of the pragmatic study design embedded in clinical practice but are still of importance. With the design, we are unable to know whether the differences observed in the pre-course and post-course material were in fact associated with participation in the ACT course. Also, as the comparisons were in the same group of spinal pain clinicians but across different patients, the differences that are found may be affected by the shift in patients. In addition, there was one assessment less in the post-course material, leaving less material to code. In addition, some assessment contained other divergences, as mentioned in the methods section. This included the presence of a translator, relatives, and additional clinicians.. This may impact the content of the assessments and therefore the analysis of themes Further, clinicians as well as patients knew the aim of the study and the fact that they were being recorded. While this is unavoidable, one need to bear in mind that it may have impacted the dialogue.

Second, some limitations were related to the analysis. Readers ought to be aware that some codes are similar in content, which may cause some overlaps in content. Further, the quantification of data by counting codes can be challenging [14]. However, since this was our only indicator of potential change, this was included despite the accompanying limitations. In addition, this decision also impacted the first part of the analysis (i.e., the thematic analysis). While the thematic analysis was indeed intended to be organic and reflexive, we applied consensus coding and a shared coding framework and thereby introduced a more positivistic and limiting approach in our work [14]. While we argue this was needed in the present project, readers ought to be mindful that the choice related to the second part of the analysis (i.e., counting codes) also impacted the first part of the analysis. Also, the analysis was focused on psychological content and related methods as perceived by two psychologists, which is likely to have had an impact on the way the material has been understood. Throughout the analysis, we attempted to focus on questions that unfolded a more psychological oriented perspective, which may indeed be a strength. However, a multidisciplinary analytical team would maybe have had a broader focus and thereby picked up on additional nuances. This also relates back to the research questions, as dialogue about symptomatology and sometimes also functioning for example at work was not coded. Likewise, it is important to note that we only used the written transcripts for the analysis. Hence, we did not evaluate things as tone of voice, irony, facial expressions, and body language, which is of course also an important part of understanding communication. Also, we were not blinded to assessment timepoints in the analysis, which can have caused an interpretation bias. Further, we did not take notion of differences between clinicians in the analysis. Some elements may therefore be addressed or employed by only one or a few clinicians. Finally, we only focused on the psychologically informed elements and not the biomedical elements in the analysis. Hence, this work does not reflect the balance between these elements nor the importance of individual designing of assessments.

## Conclusions

The purpose of this qualitative study was to explore the content clinicians’ communication with patients with spinal pain in assessment situations before and after clinicians participated in an ACT course to explore its impact on communication content. This was carried out in spinal pain clinicians who received a training course in ACT as a framework to promote assessments focusing on broader and psychological oriented themes as well as applying related psychologically informed practices. Illustrated in 11 codes and three overall themes, the content of the assessment materials did consist of important psychologically informed elements and approaches. There were positive changes in 9 of 11 codes from pre to post course. While there were large differences in how much the content differed from pre-course to post-course, there was a clear increase in around half the codes. Despite of this, there is still room for improvement. While there are several limitations and points to consider, we hope to create focus on some important elements of chronic pain assessment and hereby inspire clinicians and researchers alike to engage further in this line of work to change our assessment culture to also embed psychologically informed elements and approaches. Future research will further improve our understanding of this type of training in assessment practices.

## Data Availability

The data is not publicly available due to Danish laws on data protection.

## Abbreviations

ACT: Acceptance and Commitment Therapy
SLR: Sophie Lykkegaard Ravn (author)
TEA: Tonny Elmose Andersen (author)
